# A Case of Fatal Invasive Trichosporonosis in the Setting of Immunosuppression and Post-COVID-19 Pneumonia

**DOI:** 10.7759/cureus.35079

**Published:** 2023-02-16

**Authors:** Aaron Lopacinski, Christine Kim, Mahmoud Khreis

**Affiliations:** 1 Internal Medicine, Eastern Virginia Medical School, Norfolk, USA; 2 Infectious Disease, Eastern Virginia Medical School, Norfolk, USA

**Keywords:** non-candidal yeast, trichosporon asahii, fungal infection, immunosuppression, drug resistance, trichosporonosis, sepsis, fungemia

## Abstract

*Trichosporon asahii *is an opportunistic fungus that forms septate hyphae and pseudohyphae, resembling *Candida albicans*, and causes fungemia in susceptible individuals. Risk factors for *T. asahii* infection include immunosuppression, IV catheters, and malignancy. In the present case, a 67-year-old male with a history of renal transplant on immunosuppressive therapy was hospitalized for coronavirus disease 2019 (COVID-19) pneumonia. Despite treatment with steroids and broad initial antibiotic coverage with cefepime, doxycycline, and vancomycin, the patient underwent continual respiratory decline. His sputum culture on hospital day 10 was positive for non-candidal yeast, and despite subsequent appropriate empiric coverage with micafungin and amphotericin B, the patient continued to decline and ultimately died due to the resistance of *T. asahii *to these antifungals. This case highlights the importance of suspecting *T. asahii* as an infectious cause in patients whose cultures show non-candidal yeast and initiating appropriate antifungal treatment early in their treatment course.

## Introduction

The *Trichosporon* genus involves a group of basidiomycetous yeasts that were first described as gritty nodules in wig hair known as piedra [1]. These fungi are ubiquitously found in nature and as part of the human skin, respiratory tract, and gastrointestinal/genitourinary tracts [2-4]. They classically behave as opportunistic pathogens, and of the 50 species of *Trichosporon* described so far, only 16 have been found to be pathogenic [3]. The six species primarily associated with human infection are *Trichosporon asahii*, *Trichosporon asteroides*, *Trichosporon cutaneum*, *Trichosporon inkin*, *Trichosporon mucoides*, and *Trichosporon* *ovoides*, which have been implicated in cutaneous, pulmonary, and systemic infections [4-5].

*T. asahii*, formerly known as *Trichosporon bigelli*, is a urease-positive nonencapsulated yeast that can form hyaline septate hyphae and pseudohyphae resembling *Candida albicans* [2-3]. However, its production of cylindrical arthroconidia in tandem with its urease positivity differentiates it from *Candida* and other yeast species [3].

*Trichosporon* species rely on several virulence factors, including the yeast-to-hyphae transition, biofilm formation, enzymatic activity, and its dynamic cell wall [2]. Specifically, the production of a capsular polysaccharide, glucuronoxylomannan (GXM), protects cells from phagocytosis and provides resistance to intracellular oxidative killing. Biofilm formation also permits the fungus to grow on extracellular implants, including central venous catheters or cardiac grafts. In addition, one of the biggest risk factors for trichosporonosis is hematologic disease or malignancy, which has been reported to account for 38.9-63% of cases in various studies [4,6]. Other risk factors reported for *T. asahii* infection specifically include IV catheters, AIDS, glucocorticoid treatment, and extensive burns [5]. 

*Trichosporon* infections have become an increasingly common cause of fungemia and are now the second most common cause of yeast bloodstream infections after *Candida* species [7]. Systemic infections with the fungus tend to begin with the colonization of mucosal or cutaneous surfaces with subsequent loss of surface integrity leading to seeding of the bloodstream, often precipitated by chemotherapy or catheter infection [8]. Patients most commonly present with fever, fungal sepsis, and shock but may also manifest cutaneous lesions, pneumonia, chorioretinitis, and/or renal failure [9].

In this report, we present a case of an immunosuppressed, leukopenic renal transplant patient treated with steroids for coronavirus disease 2019 (COVID-19) pneumonia who developed fungemia. His hospital course was complicated by late targeted antifungal therapy and ultimately death, and serves as an important lesson for future patient care.

## Case presentation

**Figure 1:**
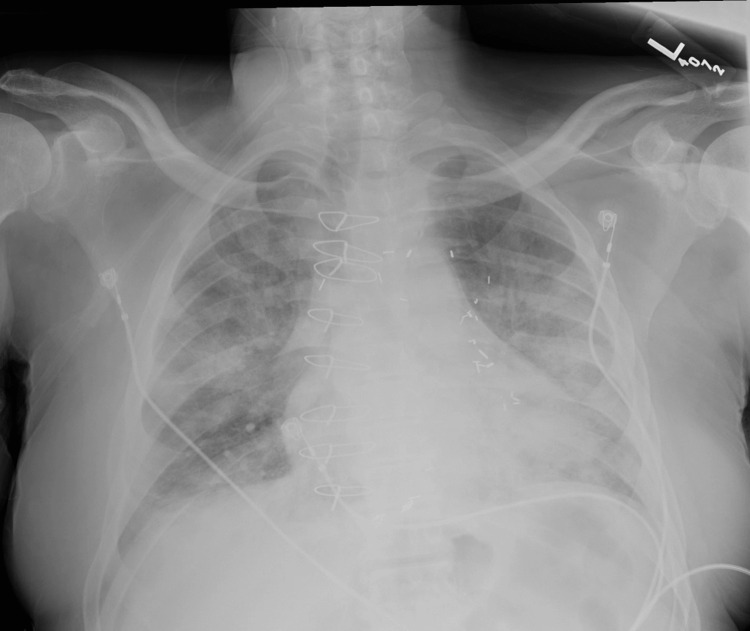
Patient's Chest X-ray on Initial Presentation Patient was determined to have bilateral pulmonary opacities most concerning in conjunction with his clinical presentation for acute respiratory distress syndrome or multifocal pneumonia.

A 67-year-old male with a history of hypertension, diabetes mellitus, coronary artery bypass graft surgery (CABG) four years ago, and chronic kidney disease with renal transplant three years prior presented with three days of shortness of breath. He was on immunosuppressive therapy with prednisone, mycophenolate, and tacrolimus, and was twice vaccinated and boosted for COVID-19. He presented hypoxic with an oxygen saturation of 88% on room air requiring high flow nasal cannula to correct shortly after his presentation. Chest x-ray showed bilateral pulmonary infiltrates (Figure 1) and bloodwork was significant for leukopenia of 1.5 with 76% polymorphonuclear neutrophils (PMNs) and 16% lymphocytes. In the emergency department, the patient was started on dexamethasone, then solumedrol and the empiric antibiotics ceftriaxone and doxycycline, for suspected multifocal community-acquired pneumonia possibly secondary to COVID-19 infection.

However, on admission to the intensive care unit, the patient's worsening condition raised concerns for multi-drug resistance, which prompted the broadening of coverage to cefepime, doxycycline, and vancomycin. His vancomycin was discontinued three days later following a negative methicillin-resistant *Staphylococcus aureus *(MRSA) nasal swab, confirmed by no MRSA growth in culture, and his cefepime and doxycycline were discontinued after completing their courses on hospital days seven and eight, respectively. However, on hospital day seven, his respiratory status further declined and he began to require pressors, prompting the insertion of central venous and arterial lines. His infectious workup at that time continued to demonstrate negative blood cultures. His sputum culture from his endotracheal tube was found to be positive and grew moderate yeast that was neither *Candida albicans* nor *Candida* *glabrata*, which was confirmed by the lab via matrix-assisted laser desorption/ionization-time of flight (MALDI-TOF) mass spectrometry (MS), but it was not speciated any further. As a result, micafungin was added to the patient's drug regimen on hospital day 11.

A temporary dialysis catheter was inserted on hospital day 12 for continuous renal replacement therapy, and transplant infectious disease was consulted on hospital day 13. They repeated blood and sputum cultures and checked a Fungitell® assay, which was elevated at 178, and a galactomannan, which was within normal limits. Due to persistent fever and concern for hospital-acquired pneumonia, vancomycin and meropenem were added to the regimen on hospital day 18. On hospital day 20, his sputum culture came back positive for pan-sensitive *Klebsiella pneumoniae* and yeast other than *C. albicans* or *C. glabrata* confirmed by MALDI-TOF MS, but with further speciation pending. Consequently, vancomycin and meropenem were discontinued and ceftriaxone was started to cover *K. pneumoniae*. His 10-day course of micafungin completed on hospital day 21, but micafungin was resumed on hospital day 25 due to lack of improvement and results of previous sputum culture resulting via MALDI-TOF MS as *T. asahii*.

Repeat sputum and blood cultures were obtained at this time, with sputum cultures demonstrating budding yeast with pseudohyphae and blood cultures showing hyphal elements that were not further characterized by the lab. His ceftriaxone was discontinued and vancomycin and meropenem were restarted on hospital day 26 due to worsening clinical status. These were switched the same day to tigecycline and ciprofloxacin out of concern for beta-lactam-induced bone marrow suppression given the patient’s continued low white blood cell count of 2.9. He was also started on granulocyte colony-stimulating factor (G-CSF), liposomal amphotericin B, and itraconazole, with voriconazole being avoided due to the patient’s renal function. This continued through hospital day 30, when the patient passed away. Blood cultures drawn previously, including samples after amphotericin B initiation, would later speciate as *T. asahii* via MALDI-TOF MS, the same species previously found in sputum cultures.

## Discussion

This case highlights the importance of early speciation and targeted, appropriate diagnosis and treatment of trichosporonosis in vulnerable populations. This patient’s early positive fungal sputum culture, which was not speciated beyond *Candida* species, could have proved useful for early initiation of antifungal therapy, especially given the presence of his numerous risk factors including immunosuppression and multiple lines. While the literature shows no consensus regarding the significance of finding *Trichosporon* species from respiratory tract secretions given its role as potentially normal flora, one study has shown that six of nine patients whose sputum grew *Trichosporon* species ultimately developed an invasive pulmonary infection [1]. The authors of this study suggested pneumonia, confirmed via increasing oxygen requirements and/or imaging findings, in absence of other identifiable pathogens should be presumed as pulmonary trichosporonosis and treated appropriately, and given the outcome of our patient, this seems like the appropriate clinical course.

Diagnosis of the disease is relatively simple, with the species being easily isolated due to its rapid growth on almost all standard fungal media [8]. Urine cultures may also be among the first to grow *Trichosporon* in disseminated disease, and its presence should not be presumed to be a contaminant in a high-risk case [4]. The species are also cross-reactive with *Cryptococcus neoformans *antigen studies, though this is only reported in 26% of cases and, thus, a negative study does not rule out the diagnosis [2,6,8]. Given the relative ease of possible diagnosis when clinical suspicion is elevated, patients can be treated empirically with appropriate antibiotic de-escalation as cultures and other studies result

The mortality prognosis of *Trichosporon* fungemia varies from 64% to 100% in several studies and thus early empiric treatment of these patients is likely essential to survival [7,10-11]. Early use of micafungin and later use of amphotericin B in our patient likely did little to help him as *Trichosporon* species show intrinsic resistance to echinocandins and poor polyene susceptibility [1,6]. Other reports of resistance vary, with some studies reporting resistance to flucytosine, fluconazole, and itraconazole [10]. General first-line therapy is voriconazole, which has been shown to improve the prognosis of hematological patients and has shown successful clearance in studies after liposomal amphotericin B failed [2,4]. Combination therapy with amphotericin B is often recommended due to disease severity despite limited activity in some studies [4]. Posaconazole has become an accepted option in cases of *Trichosporon* fungemia as well, though less information is available concerning its use compared to voriconazole. Other factors that improve survival include recovery from neutropenia and central venous catheter or other line removal [7]. G-CSF therapy as well as a reduction in steroid dosages to help resolve neutropenia are thus critical in helping resolve infection in these patients [4]. These options were restricted in our patient’s case and thus likely contributed to his mortality, but should be considered going forward in these cases, particularly in the context of high-dose steroids for COVID-19 infection.

## Conclusions

A takeaway from this case for providers is that they should be wary of positive non-*Candida* yeast in sputum cultures, particularly in the case of immunocompromised patients, and that standard empiric antifungal options, such as echinocandins, have low activity against potentially deadly *Trichosporon* infection. Early diagnosis and treatment are key, and high clinical suspicion should prompt removal of all lines if possible, restriction of immunosuppression if possible, initiation of voriconazole and amphotericin B, and initiation of G-CSF.
